# A cross-sectional National Health and Nutrition Examination survey-based study of the association between systemic immune-inflammation index and blood urea nitrogen levels in United States adolescents

**DOI:** 10.1038/s41598-024-64073-w

**Published:** 2024-06-10

**Authors:** Cheng Guo, Qinhui Cai, Yang Li, Feng Li, Kai Liu

**Affiliations:** 1 Comprehensive Pediatrics & Pulmonary and Critical Care Medicine, Kunming Children’s Hospital, No.28, Shulin Street, Kunming, 650103 Yunnan Province China; 2 Pediatric Department, Qionghai People’s Hospital, No.33, Fuhai Road, Qionghai, 571400 Hainan Province China

**Keywords:** Cross-sectional study, Adolescents, Blood urea nitrogen, Systemic Immune Inflammatory Index, Biomarkers, Endocrine system and metabolic diseases

## Abstract

Blood urea nitrogen (BUN) level is one of the commonly used indicators to assess renal function and systemic immune-inflammatory status. In the adolescent population, changes in BUN levels may be associated with a variety of factors, including physiologic dehydration, lifestyle influences such as nutritional intake, physical activity, and possible endocrine or metabolic disorders. In recent years, more and more studies have shown that BUN levels are not only a reflection of kidney function, but it may also be related to the inflammatory state of the body. The Systemic Immune Inflammatory Index (SII) is a comprehensive index that takes into account platelet counts, neutrophil and lymphocyte counts, and is thought to be effective in reflecting the body's immune status and inflammatory response. However, research on the relationship between the two, SII and BUN, remains understudied in the adolescent population. The purpose of this study was to examine the relationship between SII and BUN levels in a population of American adolescents and to further analyze the factors that influence it. We conducted a cross-sectional study using data from the National Health and Nutrition Examination Survey (NHANES) database. Using descriptive statistics, correlation analysis, and regression analysis, we explored the relationship between SII and BUN levels. We found a significant negative correlation between SII and BUN levels, with BUN levels decreasing when SII levels increased (BUN as the dependent variable and SII as the outcome variable). We performed a multiple regression analysis of this relationship, controlling for possible confounders such as gender, age, race, and BMI, and found that this negative correlation remained significant. Our findings reveal an important relationship between SII and BUN levels and provide new perspectives for understanding adolescent health.

## Introduction

In the field of medicine, an in-depth study of physiologic indicators in adolescents is key to understanding and promoting their healthy development. During adolescence, individuals experience rapid growth and changes in hormone levels in the body that have profound effects on the body's metabolism and immune system^1–5^. Of these metrics, Blood urea nitrogen (BUN) and Systemic Immune Inflammatory Index (SII) are particularly important. BUN is commonly used to assess renal health and metabolic status^6–8^, whereas SII combines neutrophil, lymphocyte, and platelet counts and is thought to provide a comprehensive picture of an individual's immune and inflammatory status^9–13^. The importance of BUN and SII is widely recognized in adult health research, but questions about these two metrics how they interact and influence each other in the adolescent population is relatively limited. The physiological characteristics of adolescence differ significantly from those of adulthood, which prevents the simple application of adult findings to adolescents^14,15^. For example, maturation of the urinary and immune systems during adolescence may affect BUN and SII levels and their correlations in different ways^16–19^. Furthermore, although several studies have examined the relationship between SII and BUN levels, the results of these studies have been inconsistent. Some studies have found a positive correlation between SII and BUN levels, whereas others have found a non-significant or negative correlation^20–24^. These contradictory results may be due to differences in research methods such as study design, sample selection, statistical methods, or different age, gender, ethnicity, lifestyle, and environmental factors that may have an impact on the relationship between SII and BUN levels.

Therefore, the aim of this study was to investigate the interconnection between two indicators, SII and BUN levels, in a population of American adolescents. Since adolescence is a critical period of growth and development, with both kidney function and the immune system in developmental flux, theoretically, as kidney function improves, BUN levels may decrease if all other conditions remain constant. However, this change interacts with changes in protein metabolism during adolescence. If protein breakdown is increased and urea production is accelerated, BUN levels may remain unchanged or elevated despite normal renal function. SII is a composite index that includes platelet count, neutrophil count, and lymphocyte count. Puberty is an important period of adaptation for the immune system, and immune cell response and regulation may be altered by hormonal changes. For example, fluctuations in sex hormones are thought to be associated with immune regulation and may affect immune cell ratios and activity, thereby affecting SII values. As immunity matures, changes in the proportion of lymphocytes in the overall leukocyte population, for example, may result in higher or lower SII. In summary, developmental changes in kidney function and the immune system during adolescence directly affect BUN and SII levels. These changes reflect the complexity and variability of physiologic maturation. Understanding the relationship between BUN and SII is of great value in revealing possible health problems during this period.

We conducted a cross-sectional study using data from the National Health and Nutrition Examination Survey (NHANES) database, a nationally representative health and nutrition survey conducted by the U.S. Centers for Disease Control and Prevention (CDC), which includes data from a variety of clinical examinations, laboratory tests, and questionnaires. Our research hypothesis was that there was a significant correlation between SII and BUN levels and that this relationship would remain after controlling for other variables.

The innovation and contribution of this study is that most of the existing studies have focused on the adult population and relatively few studies have been conducted on the specific age group of adolescents. Focusing on the adolescent population, this study explored the association between BUN and SII and further analyzed the influencing factors. The results of this study may be of potential value in the management of adolescent health and in the early identification and prevention of heart- or kidney-related chronic diseases during adolescence. We look forward to more studies in the future that will build on our study and further explore this area. We believe that this innovative finding has important implications for the study of adolescent health.

## Methods

### Study population and data sources

NHANES is a representative U.S. national population survey that uses complex, multistage, and probability sampling methods to provide a wealth of information about the nutrition and health of the general U.S. population^25^.

The Ethics Review Board of the National Center for Health Statistics (NCHS) approved the study protocol. Written informed consent was obtained from all survey participants or parents and/or guardians of individuals under 18 years of age. Visit https://www.cdc.gov/nchs/nhanes/index.htm for additional information.

**Figure 1:**
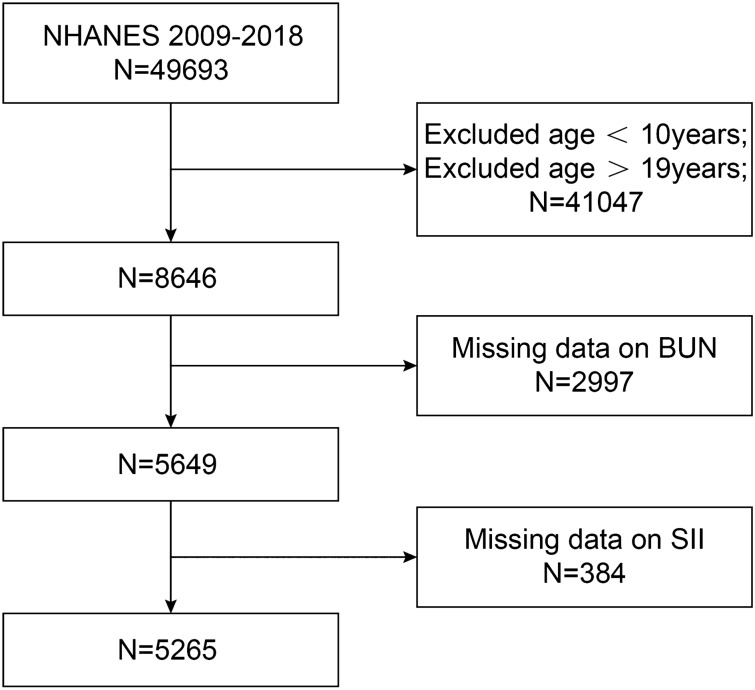
Flowchart.

Data from the NHANES 2009–2018 consecutive cycles (N = 49,693) were selected for this study to assess the relationship between BUN and SII among adolescents. First, adolescents and young adults were defined according to the World Health Organization's^26–31^. A total of 8646 eligible adolescents aged 10–19 years were included. We excluded participants with missing BUN values (N = 2997) and missing SII values (N = 384) from the eligible participants. The study ended up with 5,265 participants who met the inclusion criteria for subsequent analysis. A detailed flow chart of the participant nativity criteria is shown in Fig. 1.

### Variables in research

Venous blood was drawn from participants, and serum samples were processed, stored, and then transported to a collaborative laboratory service department for analysis. The BUN was quantitatively determined using the enzyme conductance method (DxC800 biochemistry analyzer). The complete blood count was measured by professionally trained hematological researchers using an automatic hematology analyzer (Coulter DxH 800 analyzer). Subsequently, we conducted an analysis using lymphocyte, platelet, and neutrophil counts (expressed in thousands of cells/μL). Finally, SII (1000 cells/ul) was calculated as platelet count × neutrophil count/lymphocyte count as an exposure variable^32^. The NHANES Laboratory/Medical Technician Procedure Manual (LPM) details the collection and processing of specimens in exhaustive detail.

Other covariates included sex (male or female), race (Mexican American, other Hispanic, non-Hispanic black, non-Hispanic white, or other race) and education level (less than high school, or high school), and annual household income status. Continuous covariates included BMI (body mass index), RBC (red blood cells), HGB (hemoglobin), AST (aspartate aminotransferase), ALT (alanine aminotransferase), TG (triglycerides), TC (total cholesterol), Scr (serum creatinine), GLU (serum glucose), HbA1c (glycated hemoglobin), and 25-OH-D3. Every measurement procedure is accessible to the public via the following link: https://wwwn.cdc.gov/nchs/nhanes/Default.aspx

### Methods of statistics

Every analysis used EmpowerStats software (version 4.1, http://www.empowerstats.com) and R (version 3.4.3, http://www.R-project.org). The mean by which standard deviation (S.D.) was calculated for continuous variables, while percentages were used to represent categorical variables. Analyses were conducted on the baseline characteristics of categorical and continuous variables utilizing chi-square tests and linear regression models, respectively. We categorized SII into four groups. After adjusting for potential confounders, we developed multivariate logistic regression models to explore independent associations between BUN and SII. Subgroup analyses were also conducted by gender, age, race, education, and body mass index. Three models were developed: an unadjusted model, a partially adjusted model (adjusted for age, gender, and race), and a fully adjusted model (adjusted for age, gender, race, education level, annual household income, BMI, RBC, HGB, AST, ALT, TG, TC, Scr, GLU, HbA1c, and 25-OH-D3). Values of P < 0.05 were considered to be statistically significant.

### Ethics statement

This study was reviewed and approved by the NCHS Ethics Review Board. The patients/participants provided their written informed consent to participate in The patients/participants provided their written informed consent to participate in this study.

## Results

### Population-specific baseline data

Weighted baseline characteristics of the 5265 included participants (2188 males and 2170 females) based on SII quartiles are shown in Table 1. The mean age of the included participants was 15.45 ± 2.26 years, and the mean value of BUN was 10.99 ± 3.43 mg/dL. The ranges of SII quartiles 1–4 were < 285.42, 285.42–402.16, 402.16–560.25, and ≥ 560.25. BUN levels were significantly higher in the highest quartile of SII index participants than in the lowest quartile of SII participants, and similarly, the number of people of different ages, genders, races, and educational levels was significantly higher in the highest quartile of SII index participants than in the lowest quartile of SII participants; Scr, HGB, ALT, AST, Scr, and HbA1c levels were all significantly lower than the lowest quartile of SII participants, but TG levels were significantly higher than the lowest quartile of SII participants (all P < 0.05).

**Table 1:** Baseline characteristics of the study population according to SII stratification.

Characteristics^a^	Q1 (< 285.42)	Q2 (285.42–402.16)	Q3 (402.16–560.25)	Q4 (≥ 560.25)	*P* value
	N = 1226	N = 1225	N = 1226	N = 1226	
Age (year)	15.20 ± 2.25	15.40 ± 2.23	15.42 ± 2.26	15.71 ± 2.23	< 0.0001
Gender (%)					< 0.0001
Male	62.70	57.40	46.49	40.61	
Female	37.30	42.60	53.51	59.39	
Race (%)					< 0.0001
Mexican American	14.63	13.95	16.19	16.75	
Other Hispanic	6.39	6.78	8.25	8.11	
Non-Hispanic White	49.92	58.27	54.85	53.91	
Non-Hispanic Black	20.69	12.64	11.24	9.81	
Other Races	8.36	8.35	9.47	11.43	
Education level (%)					< 0.0001
Primary school	18.01	16.30	15.16	14.83	
Junior high school	42.80	39.51	41.08	35.13	
High school	39.19	44.19	43.76	50.04	
BMI (Kg/m^2^)					< 0.0001
< 25.5	78.73	72.50	64.18	57.84	
≥ 25.5	21.27	27.50	35.82	42.16	
Annual household income (US$)					0.8892
< 25,000	26.04	24.94	26.13	26.03	
≥ 25,000	73.96	75.06	73.87	73.97	
RBC (1000cells/uL)	4.78 ± 0.43	4.79 ± 0.44	4.76 ± 0.44	4.75 ± 0.47	0.1160
HGB (g/dL)	14.01 ± 1.32	14.10 ± 1.31	13.93 ± 1.32	13.83 ± 1.48	< 0.0001
ALT (U/L)	17.91 ± 10.35	19.43 ± 15.50	19.26 ± 13.55	18.67 ± 15.02	0.0304
AST (U/L)	23.51 ± 8.60	24.07 ± 13.34	22.63 ± 7.65	22.35 ± 8.67	< 0.0001
TG (mg/dL)	88.55 ± 55.59	92.76 ± 61.72	98.88 ± 65.28	104.89 ± 68.99	< 0.0001
TC (mg/dL)	156.78 ± 28.64	157.37 ± 28.35	157.23 ± 28.62	159.55 ± 31.68	0.0912
SCR (mg/dL)	0.75 ± 0.27	0.73 ± 0.18	0.71 ± 0.17	0.72 ± 0.16	< 0.0001
GLU (mg/dL)	87.96 ± 12.78	88.89 ± 17.57	88.26 ± 11.91	88.30 ± 12.25	0.4025
SUA (mg/dL)	4.99 ± 1.18	5.06 ± 1.26	5.02 ± 1.30	5.13 ± 1.24	0.0517
HbA1c (%)	5.32 ± 0.52	5.33 ± 0.57	5.28 ± 0.41	5.30 ± 0.50	0.0521
25-OH-D_3_ (nmol/L)	60.24 ± 23.11	61.40 ± 23.95	59.72 ± 21.82	60.49 ± 23.91	0.3452
BUN (mg/dL)	11.50 ± 3.74	11.41 ± 3.85	10.96 ± 3.36	10.93 ± 3.30	< 0.0001

*SII* systemic immune-inflammation index, *BMI* body mass index, *RBC* red blood cell, *HGB* hemoglobin, *AST* aspartate aminotransferase, *ALT* alanine aminotransferase, *BUN* blood urea nitrogen, *TC* total cholesterol, *SCR* serum creatinine, *GLU* serum glucose, *SUA* serum uric acid, *HbA1c* glycosylated hemoglobin, *TG* triglycerides,

^a^Weighted means ± SD for continuous variables; weighted proportions for categorical variables.

Table 2 shows the relationship between BUN and SII. We found that the lower the BUN level, the higher the SII value in both the original and adjusted model 1. In the fully adjusted model it was found that for every unit decrease in BUN levels, SII increased by 2.29 units (β = − 2.29, 95% CI − 4.41, − 0.16). In addition, we further characterized an inverse relationship between BUN and and SII (P for trend = 0.1710).

**Table 2:** Correlation studies between BUN (mg/dL) and SII in US adolescents.

	Crude model	Model 1	Model 2
	β (95% CI) *P* value	β (95% CI) *P* value	β (95% CI) *P* value
Per 1 mg/dL BUN increase	− 4.29 (− 6.27, − 2.30) < 0.0001	− 2.82 (− 4.84, − 0.80) 0.0061	− 2.29 (− 4.41, − 0.16) 0.0351
Categories (SII)			
Quartile 1	Reference	Reference	Reference
Quartile 2	0.14 (− 0.34, 0.62) 0.5665	0.37 (− 0.12, 0.86) 0.1415	0.13 (− 0.41, 0.68) 0.6263
Quartile 3	− 0.70 (− 1.45, 0.06) 0.0724	− 0.63 (− 1.41, 0.16) 0.1189	− 0.32 (− 1.13, 0.50) 0.4478
Quartile 4	− 2.69 (− 7.10, 1.73) 0.2336	− 2.41 (− 6.98, 2.16) 0.3018	− 2.23 (− 6.88, 2.42) 0.3480
*P* for trend	− 0.57 (− 1.63, 0.50) 0.2974	− 0.46 (− 1.56, 0.64) 0.4094	− 0.81 (− 1.97, 0.35) 0.1710

Crude model: no covariates were adjusted.

Model 1: age, sex, race were adjusted.

Model 2: age, sex, race, education level, Annual household income, body mass index (BMI), RBC, HGB, ALT, AST, TG, TC, SCR, GLU, SUA, HbA1c, and 25-OH-D_3_ were adjusted.

Table 3 shows that in subgroup analyses stratified by gender, ethnicity, age group, and BMI, our results indicated that the negative association between BUN and SII was independently significant among US adolescents with a BMI ≥ 25.5 kg/m^2^ [− 4.77 (− 8.24, − 1.30)], but was not statistically significant in all models with a BMI < 25.5 kg/m^2^.

**Table 3:** Subgroup analysis.

SII		β (95% CI)	*P* for interaction
Gender			0.4915
Male	N = 2405	− 0.18 (− 2.85, 2.49)	
Female	N = 2215	− 1.55 (− 4.43, 1.33)	
Age (years)			0.8843
< 17	N = 2953	− 0.37 (− 2.83, 2.08)	
≥ 17	N = 1667	− 0.67 (− 3.84, 2.50)	
Race (%)			0.4964
Mexican American	N = 1047	− 0.52 (− 5.87, 4.83)	
Other Hispanic	N = 505	1.49 (− 6.32, 9.29)	
Non-Hispanic White	N = 1279	− 0.50 (− 3.09, 2.08)	
Non-Hispanic Black	N = 1062	2.15 (− 3.49, 7.79)	
Other races	N = 727	− 5.25 (− 11.59, 1.10)	
BMI (Kg/m^2^)			0.0079
< 25.5	N = 3058	0.88 (− 1.47, 3.23)	
≥ 25.5	N = 1562	− 4.77 (− 8.24, − 1.30)	

**Figure 2:**
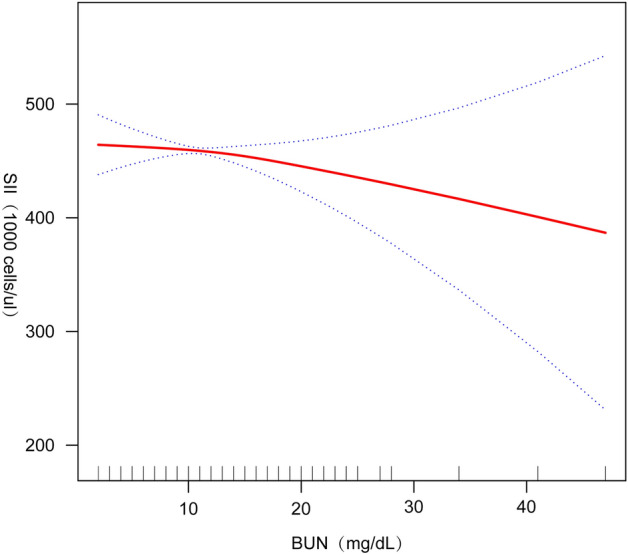
Smooth curve fitting.

**Figure 3:**
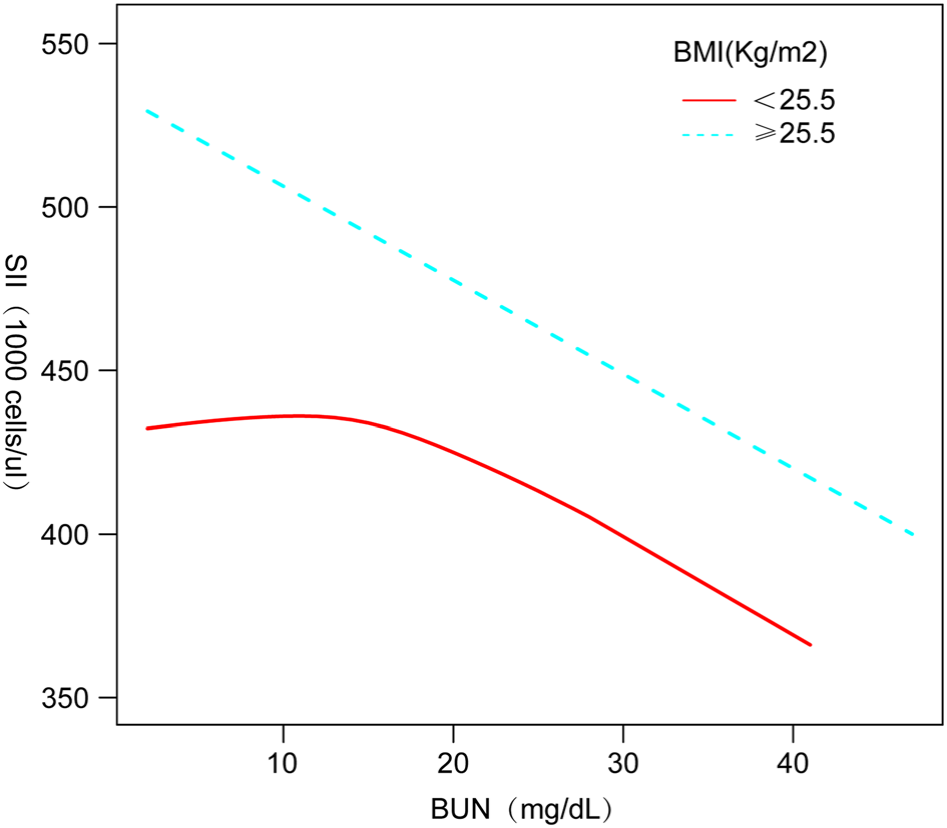
BMI Smooth curve fitting.

We performed smoothed curve fitting (Fig. 2) further in order to characterize the inverse relationship between BUN and SII. Using a two-stage linear regression model (Table 4), we found that stratified analyses by BMI revealed an inverse U-shaped curve for American adolescents with BMI < 25.5 kg/m^2^ and BMI ≥ 25.5 kg/m^2^ (Fig. 3), with an inflection point of 13 (mg/dL).

**Table 4:** Threshold effect analyses of BUN and SII in U.S. adolescents with different BMI.

BUN (mg/dL)	Adjusted β (95% CI)P value
Fitting by the standard linear model	− 6.6 (− 12.7, − 0.5) 0.034
Fitting by the two-piecewise linear model	
Inflection point	13
BUN < 13 (mg/dL)	0.9 (− 2.5, 4.2) 0.608
BUN ≥ 13 (mg/dL)	− 5.7 (− 9.8, − 1.6) 0.006
Log likelihood ratio	0.033

## Discussion

The central finding of this study is that there is a significant negative correlation between SII and BUN levels in the US adolescent population. This means that as SII increases, indicating increased inflammation and immune activity, BUN levels show a downward trend.This result not only reveals the potential importance of SII and BUN levels in adolescent health monitoring, but also highlights the complex interactions between the immune system and metabolic processes during adolescence.

First, elevated SII usually reflects the body's response to inflammation^33–36^. In adolescence, this response may be more complex, as the immune system is rapidly developing and adjusting^37–39^. For example, one study demonstrated differences in immune cell ratios and activity in adolescence compared to adults, which may influence levels of inflammatory markers^40^. Second, the decrease in BUN levels may reflect specific changes in body and kidney function in inflammatory states^41,42^, and in certain pathological states, such as severe inflammation or infection, the body may respond to external stresses by altering metabolic pathways, such as by increasing protein synthesis and decreasing catabolism, which may similarly affect BUN levels. Also, even in the presence of impaired renal function, the kidneys may partially compensate for decreased excretion of urea by increasing the excretion of other metabolic wastes (e.g., creatinine). In addition, the kidneys may regulate BUN levels by altering urea reabsorption, especially in mild or moderate renal insufficiency. This is particularly critical in the adolescent population, as they are at a critical stage of growth and development. The kidneys, as the main organs of metabolism and excretion, are particularly sensitive to hormonal changes^43^. It has been suggested that hormonal fluctuations during adolescence may affect renal function and regulation^44^. During the rapid growth phase of adolescence, the metabolic activity of an organism tends to support the establishment and development of new tissues, which typically requires more anabolic processes to meet the demands of growth. As a result, the breakdown of proteins and other key nutrients may be reduced, which helps to explain why BUN levels may be lower during this growth phase, as the primary source of urea is the breakdown of proteins. Notably, this study found that the relationship between SII and BUN remained significant even after controlling for age, gender, race, and BMI. This finding suggests that the association between SII and BUN may be driven by deeper physiological processes^8^. For example, gender differences may influence immune responses and metabolic processes in adolescents^45–47^. One study showed significant differences in immune response between adolescent females and males, which may influence their inflammatory markers^48^. In addition, the influence of lifestyle factors, such as diet and exercise, on SII and BUN cannot be ignored. In adolescents, the occurrence of dehydration may be closely related to lifestyle (e.g., physical activity, drinking habits, etc.) due to physiological and behavioral peculiarities. Dehydration leads to a decrease in body water, which increases blood viscosity and plasma osmolality. This change stimulates the kidneys to conserve water by decreasing the volume of urine, which in turn leads to higher blood urea nitrogen (BUN) concentrations. In adolescence, when renal function and metabolic status may be more active or unstable due to growth and development, dehydration may lead to more pronounced changes in these indicators. At the same time, dehydration may affect the counts and ratios of these cells, including platelets, neutrophils, and lymphocytes, through several mechanisms. First, hemoconcentration due to dehydration can artificially increase cell counts in the blood, including neutrophils and platelets, which may lead to elevated SII. Second, prolonged or severe dehydration may affect the body's immune response by affecting the cellular function and survival environment, which in turn may affect the body's immune response^49,50^.

Several studies have noted that SII tends to be elevated and positively correlated with BUN levels in chronic disease states such as chronic kidney disease or cardiovascular disease^24,51^, whereas our study observed a negative correlation between SII and BUN in a generally healthy group of adolescents. This difference may stem from the different health states of the sample groups. In chronic disease states, the inflammatory response may be more pronounced, thus affecting renal function and leading to elevated BUN. In healthy adolescents, on the other hand, lower SII may reflect lower levels of inflammation and normal renal function, so BUN is maintained at a lower level. Secondly, some studies have found that SII and BUN may be positively correlated in metabolic abnormalities conditions such as metabolic syndrome^52^, which again differs from our findings. This may be due to the fact that inflammation and metabolic abnormalities in patients with metabolic syndrome may lead to impaired renal function, which in turn affects BUN levels. In the general population of adolescents, this relationship is less pronounced due to the lack of significant metabolic disease burden.

Overall, this study emphasizes the importance of focusing on SII and BUN levels in adolescent health surveillance. It is hoped that it will provide new perspectives on adolescent health management and disease prevention, and provide a more solid foundation for further subsequent studies. Of course there are some limitations and shortcomings in our study. First, our data were obtained from the NHANES database, and although this is a nationally representative health and nutrition survey data, its data quality and reliability still need to be further verified. Second, because of the cross-sectional design of this study, we were only able to observe a correlation between SII and BUN, and could not determine whether, and what kind of causal relationship existed between them. Future studies should adopt a longitudinal study design to track individual changes over time in order to reveal causality more accurately. Finally, our sample may not have fully covered adolescents of different ethnic and socioeconomic backgrounds, which may affect the generalizability of the findings. Future studies should include a wider range of population groups to ensure broad applicability and validity of the results. Finally, our research model did not encompass the effects of factors such as diet, exercise, and sleep on SII and BUN, and future studies should include detailed information on these variables to more fully understand their relationship with SII and BUN.

In response to the limitations mentioned above, for future studies, we consider that improvements can be made in the following ways: First, in order to determine if, and what kind of causal relationship exists between SII and BUN, future studies should utilize a longitudinal research design. Second, expanding the diversity of the sample to include adolescents from different ethnic and socioeconomic backgrounds will ensure broader representation and applicability of the findings. This can be accomplished by collaborating multiple different districts for multi-center sample collection. Further, laboratory studies can be conducted to explore the interactions between immune response, metabolic regulation, and hormonal regulation at the molecular level. This can be accomplished through biochemical analysis of biological samples such as blood and urine. Finally, adolescent-specific intervention studies, such as diet and exercise programs, could be designed and implemented to assess how these interventions affect SII and BUN levels, thereby providing more specific recommendations for health improvement. With these improvements, future studies will not only overcome the limitations of existing studies, but also provide a more in-depth and comprehensive scientific basis for adolescent health surveillance and disease prevention.

## Conclusion

Overall, this study revealed a significant negative correlation between BUN levels and SII in a population of US adolescents and found that this association showed variability among adolescents with different BMIs. This finding has key implications for a deeper understanding of metabolic and immune functions during adolescence and provides new research directions for future studies in related areas. Future studies should aim to investigate the biological mechanisms behind this relationship and its possible impact on adolescent health.

## Data Availability

Publicly available datasets were analyzed in this study. This data can be found here: www.cdc.gov/nchs/nhanes/.
